# Material State Awareness for Composites Part I: Precursor Damage Analysis Using Ultrasonic Guided Coda Wave Interferometry (CWI)

**DOI:** 10.3390/ma10121436

**Published:** 2017-12-16

**Authors:** Subir Patra, Sourav Banerjee

**Affiliations:** Integrated Material Assessment and Predictive Simulation Laboratory, Department of Mechanical Engineering, University of South Carolina, Columbia, SC 29208, USA; spatra@email.sc.edu

**Keywords:** material state awareness, guided wave, coda wave interferometry, composite, precursor damage, progressive failure model

## Abstract

Detection of precursor damage followed by the quantification of the degraded material properties could lead to more accurate progressive failure models for composite materials. However, such information is not readily available. In composite materials, the precursor damages—for example matrix cracking, microcracks, voids, interlaminar pre-delamination crack joining matrix cracks, fiber micro-buckling, local fiber breakage, local debonding, etc.—are insensitive to the low-frequency ultrasonic guided-wave-based online nondestructive evaluation (NDE) or Structural Health Monitoring (SHM) (~100–~500 kHz) systems. Overcoming this barrier, in this article, an online ultrasonic technique is proposed using the coda part of the guided wave signal, which is often neglected. Although the first-arrival wave packets that contain the fundamental guided Lamb wave modes are unaltered, the coda wave packets however carry significant information about the precursor events with predictable phase shifts. The Taylor-series-based modified Coda Wave Interferometry (CWI) technique is proposed to quantify the stretch parameter to compensate the phase shifts in the coda wave as a result of precursor damage in composites. The CWI analysis was performed on five woven composite-fiber-reinforced-laminate specimens, and the precursor events were identified. Next, the precursor damage states were verified using high-frequency Scanning Acoustic Microscopy (SAM) and optical microscopy imaging.

## 1. Introduction

The early detection and quantification of embryonic precursor damage in composites are currently challenging due to lack of an online ultrasonic method. Typical precursor damages are developed in the form of matrix cracking, microcracks, voids, micro-buckling, local fiber breakage, local fiber-matrix debonding, etc. [1,2]. These damages can be visualized using off-line laboratory-based nondestructive evaluation (NDE) methods, for example, X-ray tomography [3], Scanning Acoustic Microscopy [4], Ultrasonic immersion scanning [5], etc. However, it is realized that the conventional ultrasonic guided-wave-based Structural Health Monitoring (SHM) at low frequencies (~100–~500 kHz) are not sensitive to these precursor damages, and often demands sophisticated pattern recognition algorithms for signal processing, offline. These statistical signal processing algorithms sometimes result in heavy computational burden. 

**Figure 1:**
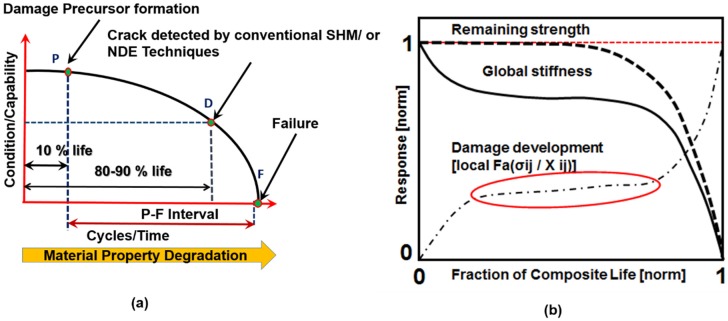
(**a**) Condition monitoring of composite structure [9,10] shows the P point when the early detection should be started; (**b**) Fatigue damage evolution in the composite material [2] shows no change in global stiffness when the incubation of embryonic damage precursor is underway.

Ultrasonic guided waves are popular for online NDE and SHM of composites [6,7]. Guided waves in a thin composite structure generate two fundamental Lamb wave modes, symmetric S_0_ and antisymmetric A_0_. In SHM, the fundamental S_0_ and A_0_ modes are analyzed to find the damages in the composite. The fundamental wave modes are useful for detecting the delamination and cracks when the physical size of the damages is comparable to the wavelengths of the propagating wave modes between the frequencies ~100–~500 kHz. However, it was found that these modes are not sensitive enough [8] to detect the precursor damage in composites. Damage precursor in composites, like micro-cracks, fiber-breakages, and crazing, starts to occur during the first 30% of the lifespan of the structure, as shown in Figure 1a. Currently, the low-frequency online NDE or SHM methods cannot detect the damages at very early stage (during the first ~30% of the life of the composites). After 80%–90% of the composite life, the interaction between the local material damages and the global structural damage is very rapid (Figure 1b). Hence, this rapid interaction causes a catastrophic failure of a structure. However, it is noted that the prelude of this event starts even before 30% of the life of the composites. So, to avoid this impending failure of the structure, it is important to implement the ‘material state awareness’ through detection of distributed precursor damages as early as possible (i.e., during the initial 30% of the lifespan of the composites). It is the objective of this article to represent the precursor state using a unique quantified parameter. Low-frequency SHM is well accepted for detecting macro scale damages in composite but is not used for precursor detection. However, in this article, to overcome the challenge in detecting the precursor damages, an online SHM method with Coda Wave Interferometry (CWI) capability is proposed. 

Symmetric S_0_ and Antisymmetric A_0_ modes are not sensitive to the small-scale distributed damages such as matrix microcracking (transverse and longitudinal cracking), fiber breakages, and local fiber-matrix debonding in composites. Thus, to enable online precursor monitoring, a few researchers suggest embedding carbon nanotube networks [11] or magnetostrictive particles [12] during the manufacturing of the composites. These methods require additional material species to be added to the material, which is often discouraged. Hence, a method is required that will not alter the constituents of the composites but detect the precursor damages online. 

Here in this paper, guided coda wave analysis is proposed. It is reported that when there is absolutely no change in the Lamb wave mode velocities, the latter part of the signal that reaches after the dominant Lamb wave modes, called “Coda wave”, is highly sensitive to the weak changes in the material. The coda wave interferometry (CWI) technique is a promising nondestructive technique, which was first used by the seismologist to detect the changes in the coda wave velocities in the earth crust during the earthquakes [13,14]. Later, this technique was successfully extended to measure the relative changes of wave velocities in the concrete due to the development of the small-scale (~mm) damages [15,16]. The frequency-dependent shifts in the coda wave velocities were estimated in the range ~150 kHz–~1 MHz [16]. Thermal effect on the coda wave velocities was estimated in [17]. Larose et al. [15] estimated the relative change (∇) in the coda wave velocity (*V*) in concrete in the order of $\nabla V/V=10^{-4}$. With precise measurement of the wave velocities, it was found that the CWI was always more accurate compared to the conventional time-of-flight measurement from the direct wave analysis. Commonly, the CWI analysis was performed using two techniques, (a) doublet [18,19,20], and (b) stretching [15,16,17,21] methods. In the doublet method, several time windows are selected in the coda part of the signal, and the shift in each time windows are calculated using the cross-correlation technique. Although promising, CWI was never used for detecting or evaluating the distributed precursor damages in carbon-fiber-composite materials. 

Composite is a heterogeneous medium designed to develop damage precursors in a distributed form. These distributed local damages interact with one another and form a fracture path when further load is applied. It was found that the coda waves are sensitive to these weak changes when they interact with the distributed damages. Perhaps, while traveling through a composite specimen, multiple interactions of the Lamb wave causes the coda signal affected by the distributed damage. It is identified that if the conventional CWI is modified for composites, the method can be a promising online tool for precursor damage detection. In this work, using a modified stretching technique, an attempt has been made for the detection of precursor damage state in woven carbon-reinforced-fiber-plastic under fatigue loading. To verify, prove, and explain the occurrence of the precursor damages in the specimens from the CWI method, benchmark studies using optical microscopy, Scanning Acoustic Microscopy (SAM), and Scanning Electron Microscopy (SEM) are conducted. 

## 2. Materials and Methods

### 2.1. Materials and Specimen Preparation

**Figure 2:**
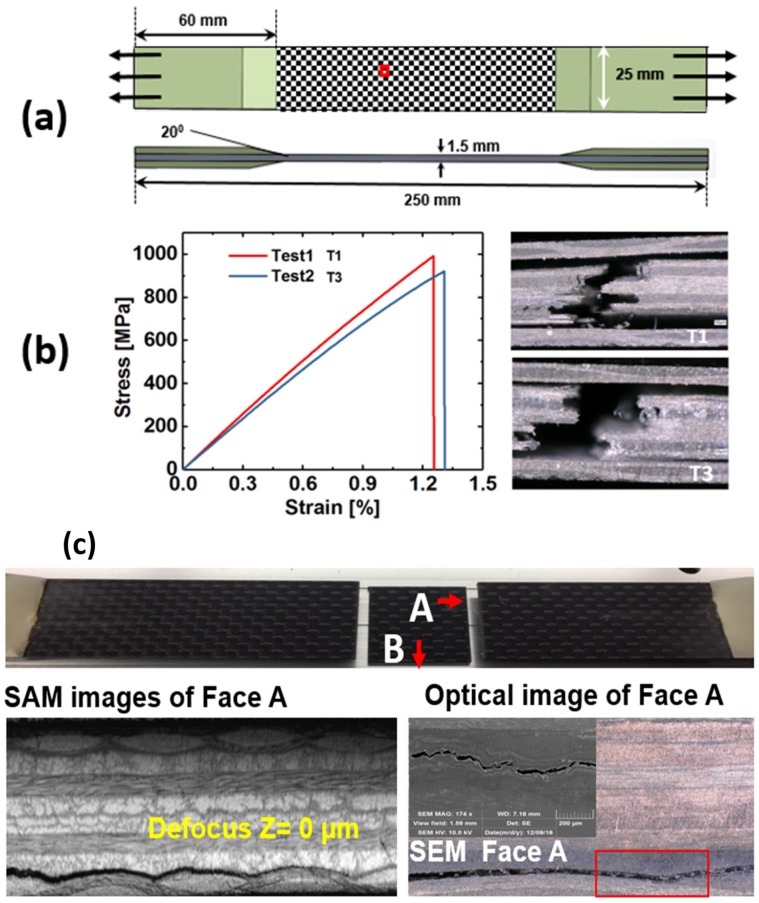
(**a**) Schematic of the specimen geometry and the material used for precursor damage experiments; (**b**) Stress-strain curves and failure images from T1 and T3 specimens; (**c**) Damages that were observed in a woven composite specimen after ~2 million cycles, delamination started after ~1 million cycles.

Four-layer woven carbon-fiber composites are used in this study (Figure 2a). The thickness of each lamina is ~280 μm. Dimension of the specimens, length and width of the tabs, and chamfer angles were chosen according to the ASTM D 3039 [22] standards. The average length, width, and thickness of the specimens were ~250 mm, ~25 mm, and ~1.5 mm, respectively. Epoxy 9340 from Loctite (48 h curing time) was used for bonding the tabs with the specimens.

Next, 7-mm-diameter PZT sensors from Steminc Steiner & Martins, Inc. (Miami, FL, USA)were mounted on the specimens using Hysol 9340 (Henkel Loctite, Stamford, CT, USA). Twelve specimens were prepared. Three specimens (T1–T3) were tested under pure tensile load (Figure 2b), one specimen (F-L) was tested under fatigue loading to estimate the maximum fatigue life of the material type. The fatigue life was intentionally marked at ~1 million cycles when an onset of delamination was first detected (Figure 2c) in the specimen F-L. However, the fatigue test was continued until the end of ~2 million cycles. From the remaining six specimens (S-A, S-B, S-C, S-D, S-E, S-H), each was tested under tensile–tensile fatigue load up to ~30% of the fatigue life, i.e., up to ~300,000 cycles. The results from specimens S-A to S-E are discussed in this article. Specimen S-H was used for the tensile test to find the remaining strength of the material at the end of 300,000 cycles.

### 2.2. Tensile Tests and Non-Accelerated Fatigue Testing

**Figure 3:**
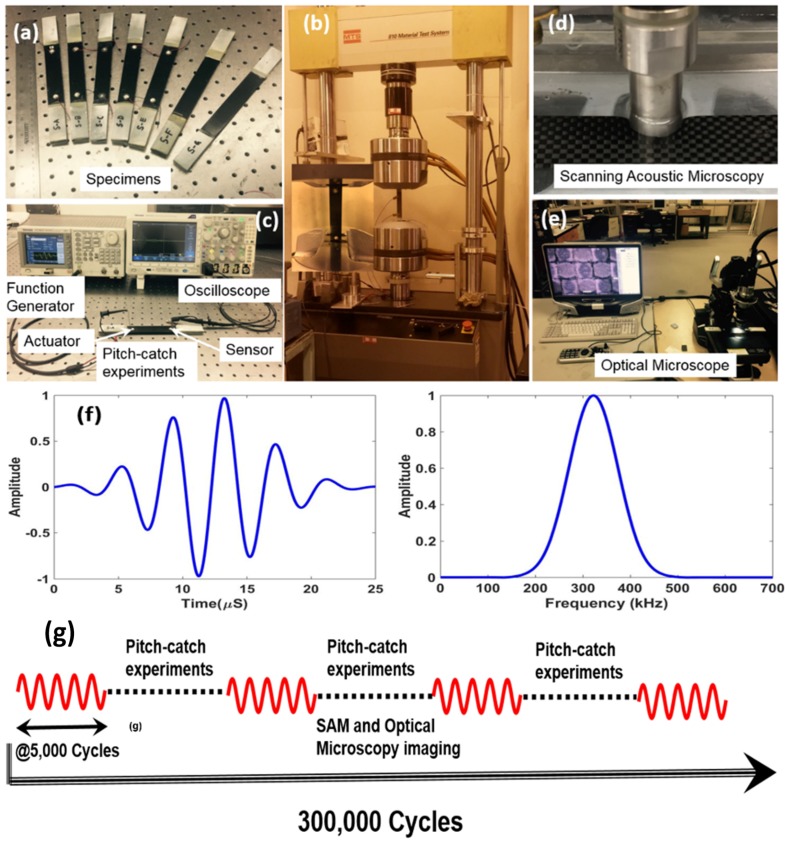
(**a**) Composite specimens that were used for fatigue testing; (**b**) Experimental set-up for fatigue testing; (**c**) Setup for pitch-catch experiments; (**d**) Scanning Acoustic Microscopy for ultrasonic inspection of the specimen; (**e**) Digital microcopy for damage inspection; (**f**) Gaussian wave signal (tone burst) used for pitch-catch experiments and its frequency transformation; (**g**) Experimental sequence.

Tensile tests were performed on the specimens T1–T3 to obtain the ultimate tensile strength of the composite material. Wire lead strain gauges 5 mm in length with size 9.5 mm× 3.5 mm were mounted on the tensile specimens using standard M-Bond 200 adhesive. Tensile load was applied with the displacement control mode at the rate of 0.03 cm/min. The average strain rate was 3.25 × 10^−5^ s^−1^. National Instrument’s data acquisition system (NI-DAQ) was used to acquire the load-strain data at the rate of 3 Hz. Figure 2b shows the test results from the T1 and T3 specimens (the specimen T2 failed accidentally at the stress level ~780 MPa and is discarded from Figure 2b). The average maximum strength of the material was ~950 MPa. Next, to study the precursor damage initiation, the remaining specimens were tested with the tensile–tensile fatigue loading on an MTS 810 machine, with loading frequency of 10 Hz, load ratio R = 0.01 (R = F_min_/F_max_), and maximum load kept ~50% of the tensile strength, i.e., 17.8 kN, up to ~300,000 cycles (Figure 3a,b). During the fatigue testing, at an interval of 5000 cycles, ultrasonic guided Lamb wave experiments (Figure 3c) were performed using piezoelectric sensors mounted on the specimens in the pitch-catch mode. S-A, S-B, S-C, and S-D were used for online precursor damage detection and were equipped with piezoelectric wafer active sensors. S-E was used for both online CWI and offline SAM and optical microscopy (Figure 3d,e) investigation. Specimen S-A was decommissioned after 300,000 cycles to perform the SAM. The specimens S-A and S-H were subjected to similar fatigue loading until 300,000 cycles. After the fatigue experiment, the specimen S-H was tested under pure tension, to find if the precursor damage state has compromised the strength of the composite material.

### 2.3. Pitch-Catch Ultrasonic Lamb Wave Experiments

Two high-frequency PZT sensors were attached to the specimens. A 5-count tone burst signal with central frequency, f_c_ = 324 kHz, was used for the actuation of the guided wave, as shown in the Figure 3f. The central frequency ~324 kHz was selected from a tuning experiment with the specimen S-A, where the fundamental antisymmetric wave mode had a maximum amplitude. Tektronix AFG3021C (25 MHz, 1-Ch Arbitrary Function Generator, Tektronix Inc., Portland, OR, USA) was used to generate the tone burst actuation at the interval of 1 ms. Peak to peak amplitude of the burst signal was set to 20 V for the wave actuation. Tektronix MDO3024 (200 MHz, 4-Ch Mixed Domain Oscilloscope, Tektronix Inc., Portland, OR, USA) was used to record the sensor signals. Sensor signals were collected at 50.0 MS/s with 10,000 data points. Online pitch-catch experiments were performed keeping the specimen on the loading machine. All benchmark studies were performed at the pristine state and after the 300,000 cycles of fatigue. Sensor signals using PZT sensors were recorded every 5000 cycles using an oscilloscope (Figure 3c) and a total of 61 data files were saved for each specimen during the 300,000-cycle experiment. 

### 2.4. Stretching Technique with Cross-Correlation

If the coda part of the wave signals (the signal that arrives after the Lamb wave modes) from two material states (1 = pristine and *k* = *k*th material state) are represented as *s*_1_(*t*) and *s_k_*(*t*), respectively, then their relation can be written as.

*s_k_*(*t*) = *s*_1_(*t*(1 + *α*)) + *d*(*t*) (1)

where, *α* is a relative stretch parameter, and *d*(*t*) is the distortion. In the stretching method, the time scale of the perturbed state signal was stretched (+ sign) or compressed (− sign) by a suitable stretch parameter value, *α* as *t_k_* = *t*(1 + *α*). A range of *α* values was selected [−value ≤ *α* ≤ value], and cross-correlation was performed between, *s_k_*[*t*(1 + *α*)] and *s*_1_(*t*). A value of *α* that maximizes the normalized cross-correlation was considered as the critical stretch parameter (*α*_*k*_) and was used to measure the relative average velocity change in the medium [16]. Item $\alpha_{k}$ is the relative change of velocity between two material states (1 and *k*).

(2) $$\mathrm{CrCr}\left(\alpha \right)=\frac{\int_{t-T/2}^{t+T/2}s_{k}\left[t\left(1+\alpha \right)\right]s_{1}\left(t\right)\mathrm{dt}}{\sqrt{\int_{t-T/2}^{t+T/2}s_{k}^{2}\left[t\left(1+\alpha \right)\right]\mathrm{dt}\int_{t-T/2}^{t+T/2}s_{1}^{2}\left(t\right)\mathrm{dt}}}$$

(3) $$\alpha_{k}=\underset{\alpha \in \Omega }{\max}\left({\mathrm{CrCr}}_{k}\left(\alpha \right)\right)$$

where *T* is the time window selected in the calculation above.

### 2.5. Taylor Series Expansion

An alternative approach to calculate $\alpha_{k}$ is presented herein. Using the Taylor series expansion of *s*_1_(*t*(1 + *α*)) up to 1st order the Equation (1) can be as follows,

(4) $$s_{k}\left(t\right)=s_{1}\left(t\right)+s_{1}^{\prime }\left(t\right)t\alpha +Higer\;order\;terms$$

As the stretch parameter, *α*, is very small, the higher order terms in the Equation (4), can be neglected. *α* is approximated as follows,

(5) $$\alpha \approx \frac{1}{N}\sum_{j=1}^{N}\frac{s_{k}(t_{j})-s_{1}(t_{j})}{s_{1}\prime (t_{j})t_{j}}$$

By employing the explicit finite difference scheme, the gradient term, $s_{1}^{\prime }\left(t\right)$ can be calculated as follows,

(6) $$s_{1}\prime (t_{j})\approx \frac{s_{1}(t_{j+1})-s_{1}(t_{j-1})}{t_{j+1}-t_{j-1}}$$

where, $t_{j+1}-t_{j-1}=2\Delta t=2/f_{s}$, $f_{s}$ is the sampling rate. Substituting Equation (6) in Equation (5), relative stretch parameter, $\alpha_{k}$ after the *k*th material state (here *k*th fatigue interval) is calculated as follows.

(7) $$\alpha_{k}\approx \frac{1}{N}\sum_{i=2}^{N+1}\frac{2[s_{k}(t_{i})-s_{1}(t_{i})]}{f_{s}t_{i}[s_{1}(t_{i+1})-s_{1}(t_{i-1})]}$$

where *N* is the total number of data points used in the calculation, $t_{i}$ is the timestamp of the *i*th data, and $f_{s}$ is the sampling frequency. This technique is computationally less expensive than the cross-correlation technique and is used to calculate the stretch parameter for the long-range signals [23], which is used in this study for the precursor damage quantification in composites.

### 2.6. Precursor Damage Growth Parameter

It was found that the change in the stretch parameter due to the coda wave velocities could be incremental in both positive and negative direction with respect to the positive time axis. Hence, instead of calculating the stretch parameter by comparing the pristine and damaged state signals, as a baseline-free method, incremental stretch parameter is calculated between two signals recorded at two consecutive states ((*k* − 1)-th state and *k*th state) as follows,

(8) $$\alpha_{k}\approx \frac{1}{n}\sum_{i=2}^{n+1}\frac{2[s_{k}(t_{i})-s_{k-1}(t_{i})]}{f_{s}t_{i}[s_{k-1}(t_{i+1})-s_{k-1}(t_{i-1})]}$$

This helps to avoid the distortion part *d*(*t*) in the Equation (1), where two very closely correlated signals in the two consecutive fatigue states were used to calculate incremental stretch parameter. Overall, the damage growth is quantified using the Precursor Damage Index (PDI) from the cumulative sum of the stretch parameters over the total duration of the fatigue life. The absolute value of the sum of the stretch parameters was defined as PDI, as written in Equation (9).

(9) $$PDI=\left|\left(\overset{N}{\underset{k=1}{\sum }}\alpha_{k}\right)\right|$$

The time window in the coda part of the signal should be selected such a way that after stretching or compressing the coda signal from the *k*th state should be identical to the coda signal from the (*k* − 1)-th state. In this analysis, a time window T_w_ of 8 μs was used and was slid over the entire coda part of the signal. The cross-correlation coefficients were calculated at the interval of 8 μs in the specimens S-A, S-B, S-C, S-D, and S-E. The best correlated windows where the cross-correlation coefficient was 1 were found between [136–296 μs], [133–290 μs], [134–299 μs], [129–292 μs], and [138–297 μs] for S-A, S-B, S-C, S-D, and S-E, respectively.

## 3. Results

### 3.1. Understanding the Stretch Parameter

**Figure 4:**
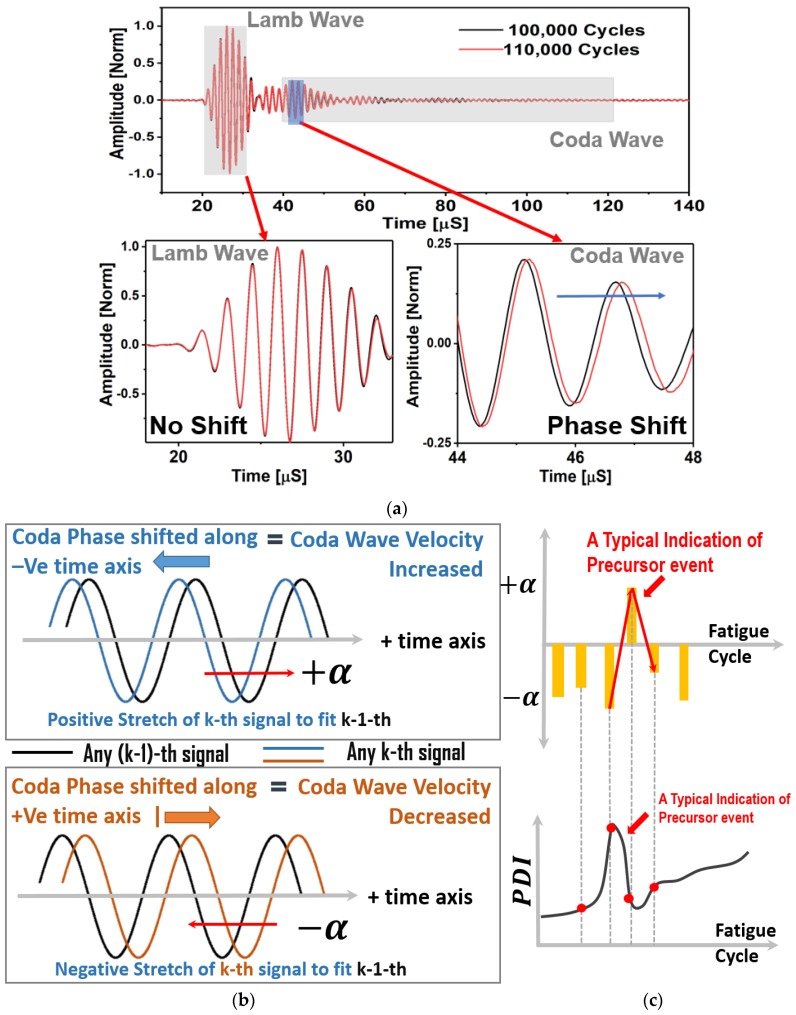
(**a**) A typical comparison between two sensor signals obtained after two consecutive material states, which shows that the first arrival of Lamb wave signals are unaffected, but the coda wave signals are time-shifted; (**b**) A conceptual schematic showing the relation between the positive and the negative stretch parameters with coda wave velocity between two consecutive material states; (**c**) A conceptual schematic showing the change in stretch parameter over the fatigue cycles and a typical scenario when the precursor damage event could be identified.

Coda part of a guided wave ultrasonic signal is affected by multiple scattering and reflection of the propagating Lamb wave modes. It was found that the coda part of the signals preserves the shape of the code wave packets between the two consecutive loading intervals. However, the phases are shifted in time. It is to be clearly noted that the signal analyzed in this article are observed during the first 30% of the lifespan of the specimens (See Materials and Methods). During this first 30% of the lifespan, it is confirmed that there were no phase shifts (Figure 4a) in the Lamb wave packets consisting of symmetric and antisymmetric wave modes. However, such shifts (Figure 4a) are evident from the coda part of the signals. The phase shifts observed in the coda part of the wave signals are independent or decoupled from the first arrival of the Lamb wave modes. This unique phenomenon in composite was not reported before and reported for the first time herein. Two sensor signals, one after 100,000 cycles of fatigue loading and another after 110,000 cycles of fatigue loading, are presented and compared in Figure 4a to prove the above claim. Phase shifts in the Lamb wave signals due to the precursor damages is zero in Figure 4a. However, the phase shifts in the coda wave signals are significant, and in fact, these shifts are very predictable. Thus, the traditional opinion about the randomness of the coda signals in the composites is incorrect. Their predictive behavior is demonstrated in the Figure 4b,c.

A conceptual schematic describing the meaning of the parameter used in this article is presented in Figure 4b,c. A few statements are deduced based on the knowledge obtained from the data and are written below:

- A positive (+) stretch parameter $\left(\alpha_{k}\right)$ is defined, when it is required to pull the coda signal from the (k) state towards the positive time axis to match the previous signal from the previous fatigue interval (k−1). This means that the $+\alpha_{k}$ is to compensate the increased coda wave velocity.
- A negative (−) stretch parameter $\left(\alpha_{k}\right)$ is defined, when it is required to push or squeeze the coda signal from the (k) state towards the negative time axis to match the previous signal from the previous fatigue interval (k−1). This means that the $-\alpha_{k}$ is to compensate the decreased coda wave velocity.
- Next, using the definition of PDI in Equation (9), it is observed that when the stretch parameter flips its sign from negative to positive or positive to negative, the PDI decreases or increases, respectively.
- It was found from the fatigue experiments that the stretch parameter is usually negative for the decreasing wave velocity, which should give rise to the PDI. However, after a sudden peak in the negative stretch parameter, the stretch parameter switches its sign to the positive, whenever the negative stretch is maximum. This makes the PDI decrease due to the increase in the coda wave velocity. Again, this is specific to the coda wave velocity only.
- Almost every time when the stretch parameter switches to positive at the end of any material state *k*, it is observed that at the end of the following state, *k* + 1 resulted inevitable negative stretch parameter. The reason for this phenomenon is explained in the Discussion section.
- The above is not applicable for the Lamb wave modes that arrive first. In case of macro-scale damage, the resulted slowness in fundamental Lamb wave modes result monotonically increasing damage index, but this is not the case reported in this article.
- It is emphasized again that the decrease in the PDI happens only and only due to the coda wave characteristics during the precursor events. A decrease in PDI is an indication of accumulated damage due to precursor in the composite which cannot be ignored and must be reported.
- It is reported herein that these unique features are found to be the pivotal in studying the precursor damage in composites using the guided coda wave.

### 3.2. Damage Growth Quantification Using PDI

**Figure 5:**
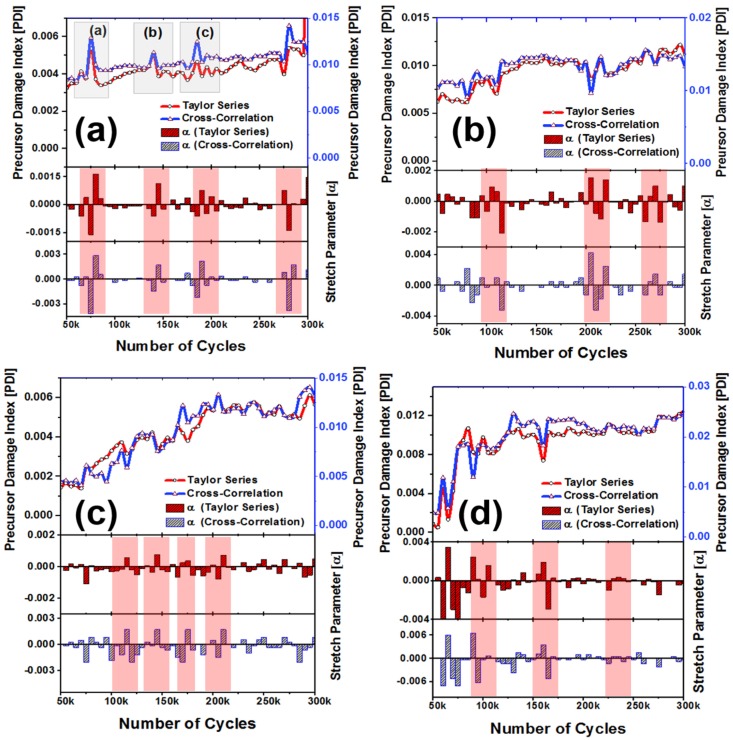
Precursor Damage Index (PDI) and stretch parameter plots for specimens, (**a**) S-A, (**b**) S-B, (**c**) S-C, and (**d**) S-D. Precursor events are marked using the red rectangles; all specimen show precursor initiation near ~120–160 k fatigue cycles.

Damage growths in five specimens S-A, S-B, S-C, S-D, and S-E were quantified with the increasing number of fatigue cycles (50,000 cycles to 300,000 cycles). The precursor damage index (PDI) was calculated employing both the cross-correlation and the Taylor series expansion technique (Figure 5). It is evident that with the help of the stretch parameter obtained from the cross-correlation and the Taylor series expansion method, overall the cumulative PDI increases with the fatigue cycles in all the four specimens, indicative of material degradation. However, few specific peaks and dips were observed in the PDI from both the methods at certain intervals, as explained in Figure 4c. Lifespan of the specimens under operation, simulated by the number of fatigue cycles associated with these fluctuations, are consistent between these two methods. These fluctuations are even consistent among all the specimens. Thus, it is evident that the PDI has indicated a physical phenomenon which is realized to be the indicators of precursor damage in composites.

## 4. Discussion

### 4.1. Explanation of PDI Data

A peak in the PDI corresponds to the decrease in the wave velocity in the guided coda wave signal (defined as negative stretch, see Figure 4b), whereas a dip in the PDI correspond to the increase in the wave velocity in the coda signal (defined as positive stretch, see Figure 4b,c). 

**Figure 6:**
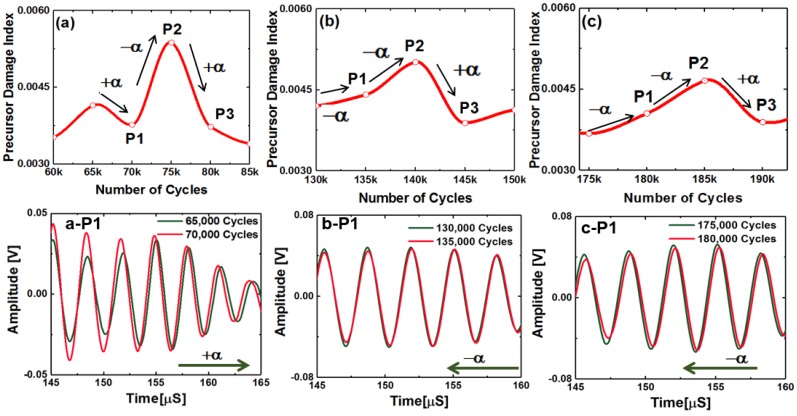

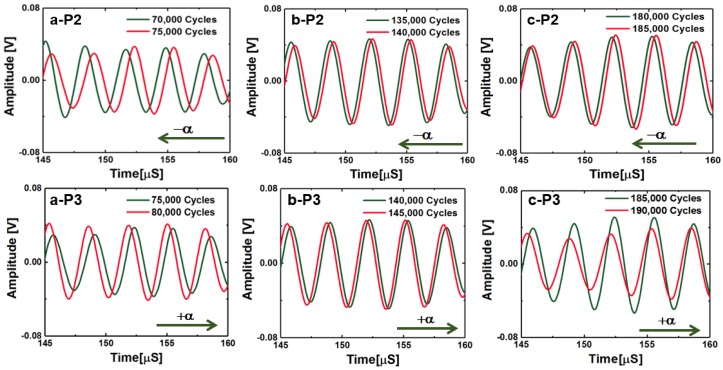
Close investigation of the peaks a, b, and c, in the PDI indicated in Figure 5: figures show the phase shifts between two consecutive coda wave signals that resulted in the peaks at **a**, **b**, and, **c** in the PDI with P1, P2, and P3 being the PDI data points, see Table 1.

From here onwards in this article, the ‘wave velocity’ is synonymous with the wave velocity of the coda wave signal, but does not represent the fundamental Lamb wave mode velocity by any means. The decrease in the wave velocity in the coda wave signal (i.e., negative stretch) is due to the distributed damages at the microscale, which led to the local degradation of the material properties and local stress concentrations, whereas, the increase in the wave velocity in the coda wave signal (i.e., positive stretch) is due to the microstructure reorientation and relaxation of the local stress concentrations. The increase and the decrease in the coda wave velocities are manifested by respective decrease and increase in the PDI. Almost every time when the stretch parameter switches to the positive at the end of any material state *k*, it is observed that the following state *k* + 1 resulted inevitable negative stretch parameter. With the gradual increase in the distributed damages, the material was degraded and was locally stressed, however, there is a limit to accommodate the local stress concentrations and suddenly the material tends to reorganize itself by relaxing the stresses. This causes an inevitable negative stretch followed by a positive stretch. Hence, the sudden decrease immediately followed by the increase in the PDI can be explained by the local formation of microscale defects and gradual healing or microstructural reorientation, which periodically takes place inside the composite specimens during the fatigue experiment. To investigate and explain this phenomenon, three peaks from the Figure 5a are selected after 75,000, 140,000, and 185,000 cycles, respectively, with their neighboring points. Slopes between the points (P1, P2, and P3) at 70,000, 75,000, and 80,000 cycles, respectively, are shown in Figure 6. The slope of the PDI curve between two consecutive fatigue intervals (at 5000 cycles) could decrease and/or increase with the loading cycles. While analyzing the PDI peak designated as (a) in Figure 5a, it can be found that the slope of the curve between P1 and P2 is positive, and slope between P2 and P3 is negative. While investigating the peaks designated as (b) and (c), similar phenomena can be observed. To calculate the stretch parameter at P2, the coda wave of the two consecutive signals (70,000 and 75,000 cycles) are compared as shown in the Figure 6a. It is observed from the figure that the phase of the coda part of the signal at the end of 75,000 fatigue cycles, leads the phase of the signal at the end of 70,000 fatigue cycles. It signifies that the average relative wave velocity in the material is decreased due to the initiation of new local damages. However, at P3 (after 80,000 cycles), the relative wave velocity is suddenly increased and can be concluded from the positive phase shift between two signals collected after 75,000 cycles and 80,000 cycles of fatigue loading. The stretch parameter at locations P1, P2, and P3 are calculated as 0.00029, −0.0042, and 0.0029, respectively, which corresponds to 0.03%, −0.42%, and 0.29% change in the average wave velocity between the two successive loading intervals. It is also interesting to note that, irrespective of the direction along time, the magnitude of the phase difference between points P2 and P3 is always higher compared to the phase difference between points P1 and P2, as evident from the Figure 6a–c. This is also in agreement with the calculated stretch parameters, respectively. Using a similar process, the percent changes in the relative wave velocity between two successive fatigue intervals were calculated at the peaks located at (a), (b), and (c) in Figure 5a and are listed below.

It is identified that whenever there has been a change in the sign of the stretch parameter, from positive to negative or from negative to positive followed by an immediate positive stretch or negative stretch value, respectively, it is a potential indication of the precursor damage in the specimen. This unique and consistent phenomenon will help devise new damage detection algorithm for online precursor damage quantification. After 300,000 cycles of fatigue loading, all the specimens were visually healthy and free from any damages or delamination(s). Hence, apparently, the strengths of the specimens are not compromised and should remain the same ~950 MPa. However, when the specimen S-H was tested under pure tensile load, which was subjected to similar loading cycles like in S-A to S-D, it failed at ~790 MPa. This concludes a ~17% decrease in total strength of the material after 30% of the life of the material due to the material degradation due to the precursor damages. 

### 4.2. Proof of Damage Development Using Optical Microscopy

**Figure 7:**
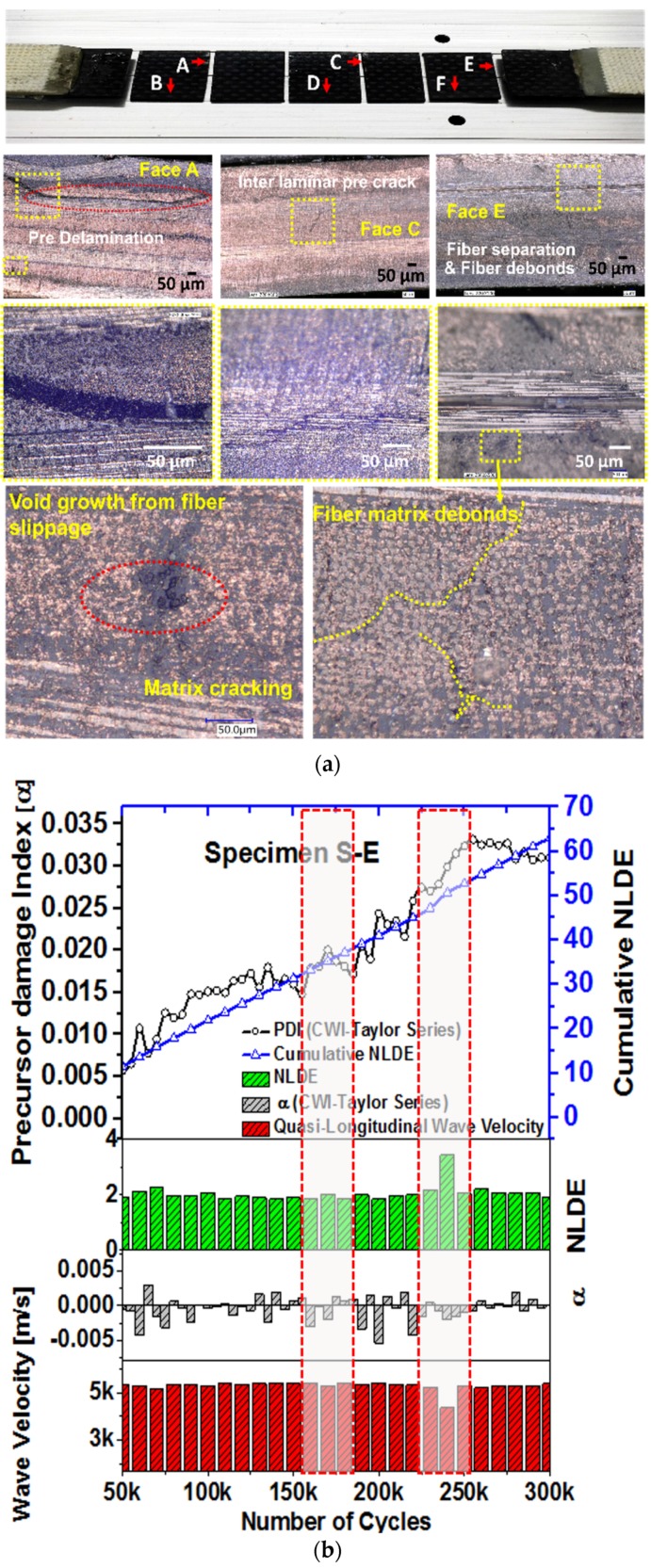
(**a**) Optical microscopy images of the decommissioned specimen S-A at the end of 300,000 cycles (**b**) Comparison of PDI with Scanning Acoustic Microscopy (SAM) analysis of the specimen S-E.

Optical microscopy imaging was performed on the composite specimens to examine the development of the micro-cracks inside the specimens. At the pristine state, very few damages were present in the form of local voids caused by manufacturing defects in the specimens (max. size ~±5 µm). However, it is evident from the microscopy images that the density of the microstructural damages increased due to the fatigue loading. Matrix cracking, fiber breakage, and localized inter-laminar delamination were observed at the end of the ~160,000 and ~300,000 cycles. The average size of the matrix-cracks was observed close to ~224 μm. Large-scale damages such as edge delamination were not observed in the specimens. To investigate the development of the precursor damages across the width, the specimen S-A was decommissioned and was cut into three pieces (Figure 7a) after 300,000 cycles of fatigue loading. Pre-delamination, fiber separation, and fiber disbond, voids from fiber slippage, and interlaminar delamination crack joining two adjacent matrix cracks are evident in the specimen S-A (Figure 7a). It is evident that the precursor damages were initiated.

### 4.3. Damage Characterization Using SAM

Scanning Acoustic Microscopy (SAM) was performed on the specimen to investigate the damage developments on the surface as well as inside the specimens, which were not accessible by the Micro-optical microscopy. SAM method is previously described elsewhere and the details are omitted herein. TSAM is a laboratory-based nondestructive method. SAM was performed on the specimen S-E at every ~30,000 cycles until 300,000 cycles. Specimen S-E was also simultaneously investigated using the PDI analysis at every 5000 cycles to have a comparative study. To investigate the status of the specimens S-A to S-D, S-A was decommissioned and was investigated using SAM after 300,000 cycles.

#### 4.3.1. SAM Method Showing Damage Growth in the Specimen S-E

**Figure 8:**
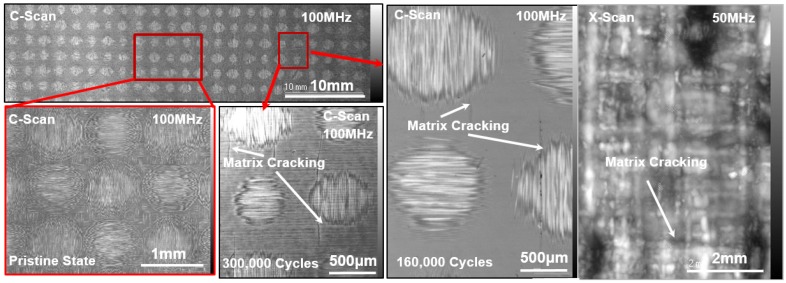
Scanning Acoustic Microscopy images at pristine state, 160,000 cycles, and 300,000 cycles.

S-E was investigated using both the CWI and the SAM methods. SAM was performed using high resolution ~50 MHz and ~100 MHz ultrasonic transducers at three defocused distances. The longitudinal wave velocities at every pixel point on the scanning areas were calculated using a method previously described elsewhere [3]. A cumulative nonlocal damage entropy (NLDE) was calculated from the SAM data and are presented side by side with the precursor damage index (PDI) obtained from the coda wave analysis (Figure 7b). A monotonically increasing NLDE with a sudden NLDE peak (~33% increase) is the indication of damage event. Two gray blocks (after ~160,000 and after ~240,000) marked in Figure 7b where the precursor damage index indicated the state of precursor damage initiation. Similar indications were also obtained from the SAM. Figure 8 shows the comparative images obtained from the SAM at the pristine state, after 160,000 cycles, and after 300,000 cycles. Matrix cracking and debonding are evident from the images. 

#### 4.3.2. SAM on the Decommissioned Specimen S-A

After the specimen S-A is decommissioned, SAM was performed using high resolution ~100 MHz ultrasonic transducers, as shown in Figure 8. Matrix cracking was clearly visible on the surface of the specimens. A couple of pre-delamination sites were also observed (Figure S1). Additionally, degraded materials properties were observed beneath the pre-delamination site. Multiple immature interlaminar delamination tracks were observed joining two matrix cracks or the tracks from the matrix-fiber disbonds. Together, it is concluded that the precursor damages are initiated in the composite specimens during as early as 30% of the life of the material. Using online CWI analysis such precursor damage events can be identified. A precursor damaged state can compromise ~17% of the ultimate strength of the material. 

## 5. Conclusions

The objective of this article was to device and prove the applicability of a reliable online precursor damage detection method. This was achieved by analyzing the coda part of the guided wave signals which are usually discarded in the conventional damage detection methods used in SHM. The proposed modified coda wave interferometry (CWI) created an opportunity to reliably detect precursor damage state in the materials. The statement is validated via multiple benchmark studies that show the actual state of the materials through images. In this work, fiber composite specimens were tested under high-cycle-low-load (HCLL) fatigue loading to develop progressive damage inside the specimen within their 30% of life calculated to be ~300,000 cycles. The modified CWI technique based on the stretching method was used for the first time for damage detection and quantification in the composite material under fatigue loading. It is identified that whenever there has been a change in the sign of the stretch parameter (in the coda wave) from positive to negative or from negative to positive followed by immediate positive stretch or negative stretch, respectively, it is an indication of the precursor damage in the specimen. 

## Figures and Tables

**Table 1:** Percent change in relative wave velocity.

	P1	P2	P3
Figure 6a	0.03%	−0.42%	0.29%
Figure 6b	−0.02%	−0.15%	0.17%
Figure 6c	−0.07%	−0.21%	0.22%

## Supplementary Materials

The following are available online at www.mdpi.com/1996-1944/10/12/1436/s1. Figure S1: Scanning Acoustic Microscopy (SAM) images from the decommissioned specimen S-A after 300,000 cycles of fatigue loading to show the precursor damages have actually initiated in the specimen.
