# Supplementary material for: Material State Awareness for Composites Part I: Precursor Damage Analysis Using Ultrasonic Guided Coda Wave Interferometry (CWI)
Source: Materials (Basel). 2017 Dec 16;10(12):1436. doi: 10.3390/ma10121436 (PMC5744371; doi:10.3390/ma10121436)
Supplement: Supplementary file 1 [file materials-10-01436-s001.pdf]

Supplementary Materials

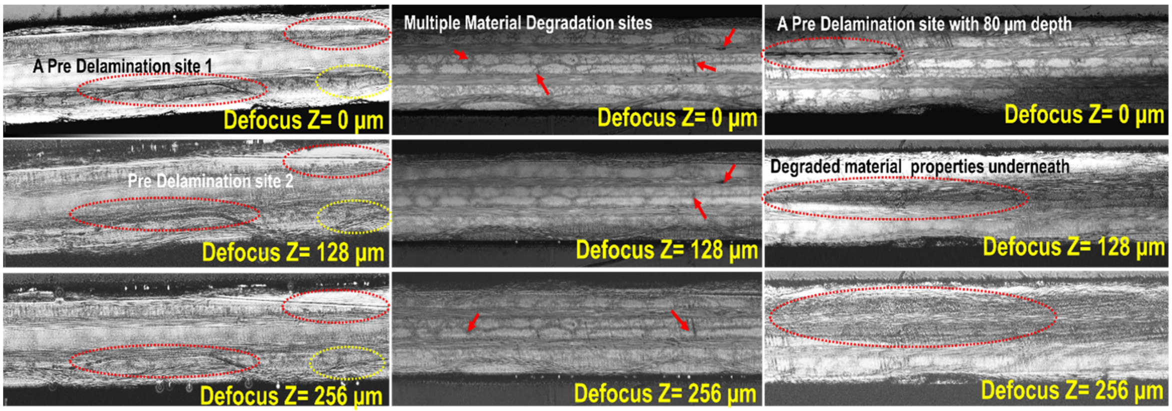

Figure S1. Scanning Acoustic Microscopy (SAM) images from the decommissioned specimen S-A after 300,000 cycles of fatigue loading to show the precursor damages have actually initiated in the specimen.
